# The clinical application potential assessment of the Deepseek-R1 large language model in lung cancer

**DOI:** 10.3389/fonc.2025.1601529

**Published:** 2025-09-02

**Authors:** Xiaowan Xu, Zhibo Liu, Shihao Zhou, Baoyan Ji, Deyan Fan, Zijuan Yang, Hongli Chen, Xiuli Yang, Mengru Guan

**Affiliations:** ^1^ The Graduate School of Qinghai University, Xining, Qinghai, China; ^2^ The Department of Oncology, Qinghai Red Cross Hospital, Xining, Qinghai, China

**Keywords:** lung cancer diagnosis, artificial intelligence, large language models, Deepseek-R1 model application, lung cancer

## Abstract

**Background:**

This study evaluates the clinical potential of the large language model Deepseek-R1 in the diagnosis and treatment of lung cancer, with a specific focus on its ability to assist junior oncologists. The research systematically assesses the model’s performance in terms of diagnostic accuracy, consistency of treatment recommendations, and reliability in clinical decision-making.

**Methods:**

A total of 320 patients newly diagnosed with lung cancer were included in this retrospective study. Twenty-six structured clinical questions were designed based on international diagnostic and treatment guidelines. These questions addressed three key domains: basic medical knowledge, complex clinical decision-making, and ethical judgment. All patient data were anonymized before being entered into the Deepseek-R1 model. The model’s responses, along with those generated by five junior oncologists with no more than three years of clinical experience, were independently assessed by senior oncologists with over ten years of experience. A double-blind evaluation protocol was implemented to reduce potential assessment bias. Inter-rater agreement was quantified using Cohen’s Kappa coefficient.

**Results:**

In the categories of basic knowledge, advanced clinical decisions, and ethical questions, Deepseek-R1 achieved average accuracy rates of 92.3%, 87.5%, and 85.1%, respectively. These rates were significantly higher than those of junior oncologists, whose accuracy rates were 80.4%, 72.8%, and 70.2%, respectively (P < 0.05). In a sample of 256 cases evaluated formally, Deepseek-R1’s overall diagnostic accuracy was 94.6%, compared to 78.9% for junior oncologists (P < 0.05). In a longitudinal assessment of 40 cases with disease progression, the model demonstrated high consistency in updating its recommendations. Logical errors were more frequent among junior oncologists, while ethical risks appeared more commonly in the model-generated responses (44% vs. 21.9%).

**Conclusion:**

Deepseek-R1 significantly outperformed junior oncologists in terms of diagnostic accuracy and treatment decision-making, particularly in complex and dynamic clinical situations. While limitations remain in its ethical reasoning, the model holds substantial potential for supporting junior physicians, contributing to multidisciplinary discussions, and optimizing treatment pathways.

## Introduction

Over the past decade, medical artificial intelligence (AI) research has primarily focused on enhancing diagnostic accuracy by utilizing molecular, genomic, and radiological datasets to drive machine learning-based interpretations of imaging anomalies. These applications include tasks such as re-stratifying skin lesions through dermatological images and distinguishing between neoplastic and non-neoplastic colorectal polyps during colonoscopy (1, 2). A common feature of these applications is their improvement in diagnostic precision; however, they still rely on direct input from medical experts and are often limited to a specific group of specialized professionals. In recent years, with technological advancements, large language models (LLMs) have emerged as pre-trained foundational models. Due to their ability to self-supervise learning on vast amounts of unstructured text, these models have demonstrated strong transfer capabilities across various tasks. Typically, these models undergo two stages of training: initial self-supervised pretraining using large-scale unstructured text data, followed by supervised fine-tuning for domain-specific tasks (e.g., medical question-answering datasets). In some cases, they exhibit notable transfer abilities in tasks like medical question answering and clinical reasoning, even with few-shot or zero-shot learning (3–8). The diagnosis and treatment of lung cancer involve a complex decision-making process, requiring multidisciplinary collaboration, image interpretation, genetic analysis, and personalized medication. Consequently, the process from the initial consultation to the lung cancer diagnosis is intricate and challenging, especially for physicians in the early stages of their careers. With the rapid advancement of AI technology, its applications have expanded across multiple fields, including society, arts, science, and medicine, particularly in image analysis. In November 2022, OpenAI released the large language model ChatGPT, based on GPT-3.5 and GPT-4. This model quickly gained widespread use across various domains (9). Trained on vast internet text, ChatGPT can generate human-like text responses and offers an easy-to-use interface for interaction in natural language. Its applications span numerous medical scenarios, including diagnostic assistance, speech recognition, transcription technology, and big data analysis. Recent studies have demonstrated the significant potential of GPT models in clinical settings, such as diagnosing geriatric pancreatitis, developing cardiovascular disease prevention strategies, and making breast cancer screening recommendations (10–12). In the management of gastroesophageal reflux disease (GERD), 93% of GPT-generated suggestions were considered “appropriate” by experts (13). In oncology, GPT has also shown considerable practical value: in lung cancer diagnosis, lesion detection accuracy reached 98.6% (8); in colorectal cancer tasks, including radiotherapy planning, pain management, and intravenous therapy, it performed effectively (14); and in thyroid cancer management, treatment recommendations based on NCCN guidelines were correct 86.8% of the time (15). These findings suggest that future large language models may become essential tools in the diagnostic and treatment processes of diseases, particularly in oncology. In this context, the recent release of the domestic large language model Deepseek-R1 in China has garnered considerable attention (16). Based on the Transformer architecture and optimized through human feedback reinforcement learning (RLHF), Deepseek-R1 excels in language understanding, multi-turn interactions, and contextual retention. Currently, the model primarily processes text inputs and does not have native multimodal understanding capabilities. However, when paired with third-party OCR tools to extract text from images, Deepseek-R1 can process text derived from images, thereby expanding its potential in scenarios that combine text and image data. Thus, for tasks such as medical image-assisted interpretation, the model’s effectiveness still relies on external image processing components. Despite this, no studies have systematically evaluated the clinical performance of Deepseek-R1 in the lung cancer diagnostic and treatment process. This study is the first to propose integrating the domestic large language model Deepseek-R1 into simulated lung cancer diagnosis and treatment tasks. By establishing structured, phased input tasks, this study aims to systematically evaluate the model’s diagnostic accuracy, consistency of treatment recommendations, and ability to adapt to ethical considerations across various domains, including basic medical knowledge, complex clinical decision-making, and ethical judgments. The model’s performance will be compared to that of junior oncologists, focusing on differences in clinical reasoning. By simulating disease progression and continuous decision-making chains, this study will explore whether the model can effectively assist young doctors in improving their diagnostic and treatment capabilities and optimizing decision-making pathways.

## Materials and methods

### Study design and patient enrollment

**Figure 1:**
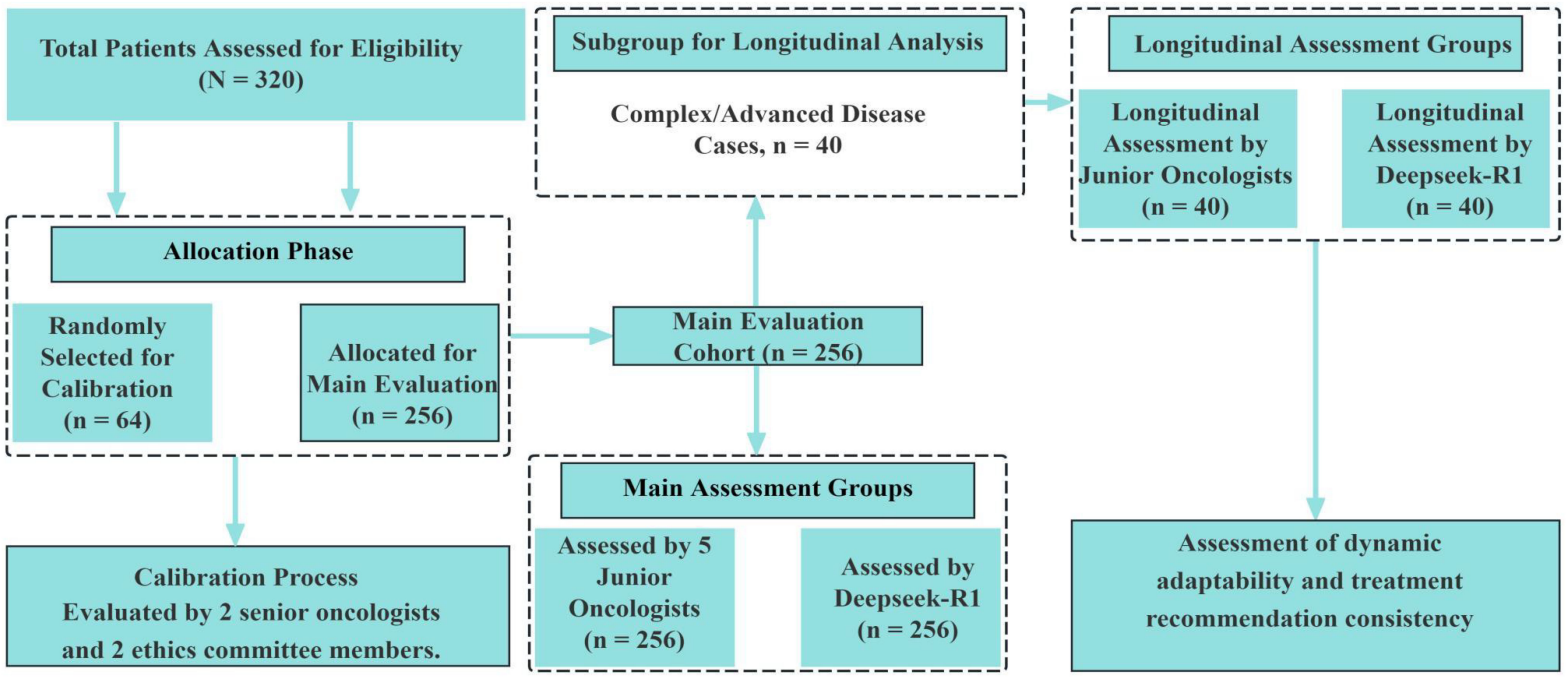
Diagram of recruitment and participation.

This retrospective study enrolled patients who were newly diagnosed with lung cancer between January 2020 and December 2024. A total of 320 eligible patients were included. Among these, 40 cases (12.5%) were classified as complex, according to predefined clinical criteria such as multiple organ metastases, rare pathological subtypes, or disease progression during treatment. These complex cases were identified based on clinical features rather than statistical sampling, in order to reflect the actual clinical distribution. All diagnoses were confirmed by senior oncologists with a minimum of ten years of clinical experience. The completeness of clinical data—including medical history, imaging results, histopathological reports, and genetic testing—was verified for all patients. Inclusion criteria were: (1) age ≥18 years; (2) newly diagnosed without prior treatment; and (3) complete and traceable clinical records. Patients were excluded if they had concurrent malignancies or if data loss exceeded 20%.To enhance the generalizability of the results, 40 complex cases were deliberately retained. These included patients with multiple organ metastases (n = 15), rare pathological subtypes (e.g., pulmonary sarcomatoid carcinoma or ALK-negative ROS1 fusion, n = 10), and those exhibiting disease progression during treatment (n = 15).All patient data were de-identified to protect personal privacy. To ensure both representativeness and randomization, all 320 patients were assigned sequential manual identification codes (P001 to P320) at the time of enrollment. A total of 64 cases (20%) were then randomly selected as the calibration sample using a manual blind draw without computational assistance. This sampling process was independently conducted by two researchers, each blinded to the clinical content of the cases. Sampling results were compared and reconciled to ensure consistency and reproducibility. To minimize evaluation bias, all responses generated by the Deepseek-R1 model and by junior oncologists were anonymized and randomly ordered prior to expert review. The expert reviewers remained blinded to the origin of each response during evaluation (see **Figure 1**).

### Clinical question design

A total of 26 structured questions were developed based on the National Comprehensive Cancer Network (NCCN) guidelines for lung cancer diagnosis and treatment and commonly encountered clinical challenges (see **Table 1**). The questions were grouped into three categories: 1. Fundamental Group (6 items): This section assessed knowledge of lung cancer definitions, common risk factors, CT-based anatomical localization, and basic diagnostic and treatment procedures. 2. Advanced Group (15 items): This section addressed complex decision-making tasks, such as subtype differentiation (e.g., adenocarcinoma vs. small cell carcinoma), application of TNM staging, selection of targeted therapies based on genetic mutations (e.g., EGFR/ALK), resistance management, and multidisciplinary consultation (e.g., neurosurgery for brain metastases). 3. Ethical Group (5 items): Topics included prognosis communication (e.g., 5-year survival explanation), fairness in clinical trial recommendations, genomic data privacy protection, and transparent error correction.

**Table 1:** Baseline characteristics of the 320 Patients.

Variable	Statistics
Age, years	64 (56–72)
Gender(n%)	
Male	218 (68.1)
Female	102 (31.9)
Smoking(n%)	
YES	190 (59.4)
NO	130 (40.6)
Pathological type(n%)	
Adenocarcinoma	205 (64.1)
Squamous carcinoma	72 (22.5)
Small cell carcinoma	31 (9.7)
Rare types	12 (3.8)
TNM stage at diagnosis(n%)	
I–II	84 (26.3)
III	94 (29.4)
IV	142 (44.3)
EGFR mutation positive(n%)	76 (23.8)
ALK rearrangement positive(n%)	18 (5.6)
PD-L1 expression >1%(n%)	126 (39.4)

### Data processing and model interaction

Before inputting into the locally deployed Deepseek-R1-7B model (~7 billion parameters), all patient data were standardized and de-identified. The model operated in a Linux environment with a single NVIDIA RTX 4090 GPU (24 GB VRAM), CUDA 11.8, and PyTorch 2.1. All model interactions used the official API without fine-tuning or customization, ensuring consistency with the default open-source inference configuration. Patient data were converted into a standardized format, including unified field naming, terminology normalization, and anonymization. The data were encapsulated in structured JSON files (see Appendix A), including demographics, medical history, imaging data, pathology, and genetic results. Each query consisted of one patient case and one question. Default parameters were applied, with a maximum output length of 2048 tokens to balance completeness and relevance. The model was accessed via the local deployment. A unified prompt template was used, integrating structured summaries of medical history, imaging, pathology, and genetics. Original imaging data were directly input into the model. However, radiological and pathological findings were standardized into text by clinicians or extracted via OCR tools. These text descriptions were embedded in the structured prompts (see Appendix B). As a result, all model inputs were text-based, ensuring consistent format and interpretability.

### Evaluation procedure and scoring criteria

After data de-identification, patient cases were sequentially entered into the Deepseek-R1 model to generate responses. At the same time, five junior oncologists (≤3 years of experience) independently responded to the same set of questions. Sixty-four cases (20%) were randomly selected as the calibration sample. These cases were excluded from the final model performance analysis, as their scoring process allowed structured consensus discussions, which differed methodologically from the formal evaluation. Two senior oncologists and two ethics committee members evaluated these calibration cases using a standardized scoring manual: 1. Fully Accurate (3 points): The response fully adhered to international guidelines (e.g., NCCN), showed coherent logic, and included all essential clinical information. 2. Partially Correct (2 points): The response was generally correct but included minor inaccuracies (e.g., incorrect dosage) or omissions (e.g., lack of comorbidity adjustment). 3.Incorrect (1 point): The response included major errors (e.g., incorrect staging or contraindicated treatments) or provided misleading guidance. Inter-rater agreement was assessed using the Kappa statistic. Discrepancies were resolved through expert panel discussions, and the scoring criteria were refined accordingly. The remaining 256 cases were evaluated under a double-blind protocol. The responses were classified as follows: 1. Correct: The response aligned with core guideline recommendations and maintained internal consistency. 2. Incorrect: The response included critical errors or breached ethical principles. For the 40 cases with disease progression, data were input into the model step by step—baseline diagnosis, treatment, and progression phases—to assess the model’s adaptability to dynamic clinical changes.

### Performance metrics and statistical analysis

The performance of Deepseek-R1 was compared with that of junior oncologists using the following metrics: (1) Accuracy: The percentage of correct answers in the Basic and Advanced groups relative to the total number of answers; (2) Compliance: The percentage of responses in the Ethics group that adhered to medical accuracy and ethical standards. An error type analysis was also performed, categorizing errors as knowledge-based errors (e.g., staging confusion), logical biases (e.g., overlooking comorbidity impacts), or ethical risks (e.g., absolute survival rate statements), and the proportion of each error type was calculated.

### Statistical analysis

All statistical analyses were performed using SPSS version 27.0. Descriptive statistics were used to summarize the baseline characteristics of the patient cohort. Categorical variables were reported as frequencies and percentages. Pearson’s chi-squared test was used to compare response accuracy between the model and junior oncologists in the Basic and Advanced question groups. A two-tailed p-value <0.05 was considered statistically significant. Responses in the Ethics question group were manually scored according to the predefined scoring manual. Inter-rater reliability was assessed using Cohen’s Kappa coefficients, calculated separately for the clinician scoring group and the ethics review panel. A Kappa value >0.75 was considered to reflect good agreement, whereas values between 0.60 and 0.75 indicated moderate agreement. Fisher’s exact test was used to compare the distribution of error types between groups, providing greater accuracy for small-sample categorical data.

## Results

**Figure 2:**
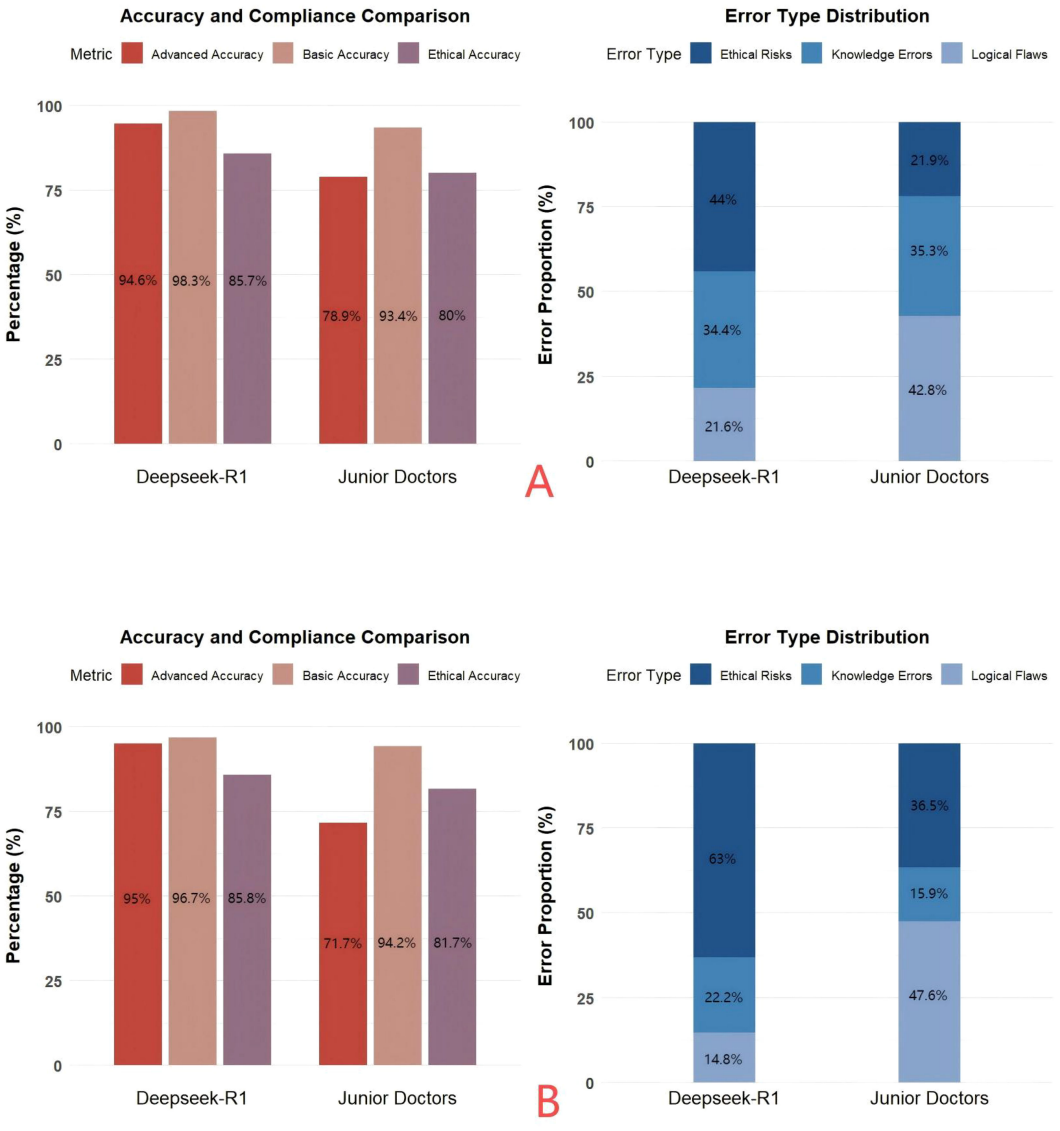
**(A)** A comparative analysis of the accuracy and error types in answering three types of questions from 256 patients by Deepseek and junior physicians. **(B)** A comparative analysis of the accuracy and error types in answering questions from 40 complex patients in the disease progression stage by Deepseek and junior physicians.

**Figure 3:**
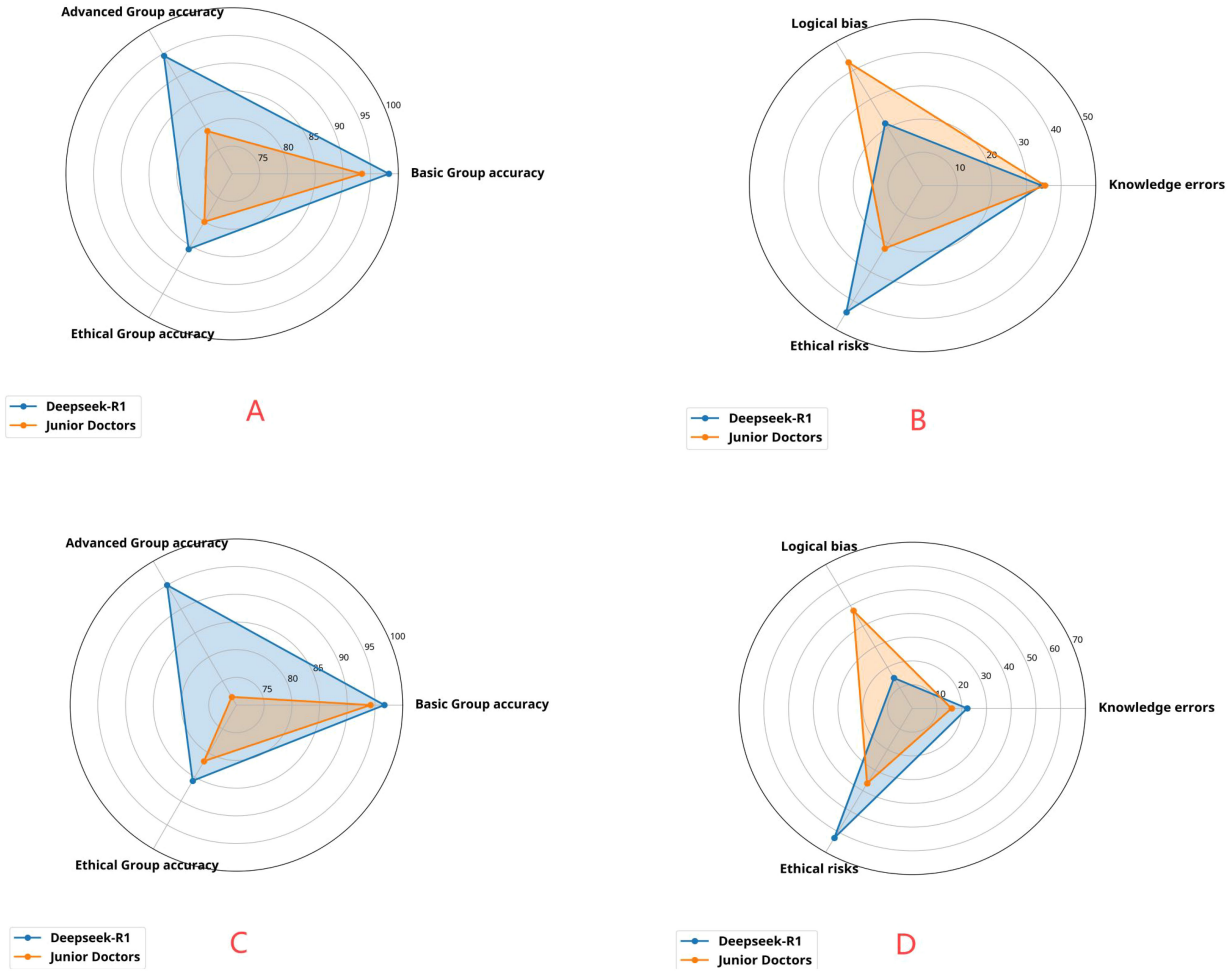
**(A)** Distribution of advanced-level accuracy, basic-level accuracy, and ethical-level accuracy of Deepseek and junior physicians in answering three types of questions from 256 patients. **(B)** Distribution of logical bias, knowledge errors, and ethical risks in the responses of Deepseek and junior physicians to three types of questions from 256 patients. **(C)** Distribution of advanced-level accuracy, basic-level accuracy, and ethical-level accuracy of Deepseek and junior physicians in answering questions from 40 complex patients at the disease progression stage. **(D)** Distribution of logical bias, knowledge errors, and ethical risks in the responses of Deepseek and junior physicians to questions from 40 complex patients at the disease progression stage.

This study included data from 320 patients diagnosed with lung cancer. The data were standardized and strictly anonymized. **Table 1** shows the baseline characteristics of the patients, including the distribution of gender, age, pathological type, TNM stage, and common genetic mutations. To ensure consistent scoring standards, dual-group scoring was initially performed, and consistency was assessed using 64 calibration samples. **Table 2** shows that the Kappa value for inter-rater reliability was 0.613 (SE = 0.088) for the oncology group and 0.669 (SE = 0.087) for the ethics review group, indicating moderate consistency in both cases. This indicates that the scoring system has good internal consistency. Given the small difference in Kappa values and the overlapping standard errors, significance testing was not performed, and no subjective judgment on the quality of consistency was made. **Table 3** compares the performance of Deepseek-R1 with that of five less experienced oncologists in completing 26 task questions across 256 formal samples. The results show that Deepseek-R1 outperformed the oncologists in all three categories: basic, advanced, and ethical questions. In the basic group, Deepseek-R1 achieved an accuracy of 98.3% (95% CI: 97.5% - 98.8%), compared to 93.4% (95% CI: 92.1% - 94.6%) for the oncologists. In the advanced group, Deepseek-R1 reached 94.6% (95% CI: 93.8% - 95.3%) versus 78.9% (95% CI: 77.6% - 80.2%) for the oncologists. In the ethics group, Deepseek-R1 achieved 85.7% (95% CI: 83.7% - 87.5%), while the oncologists achieved 80.0% (95% CI: 77.7% - 82.1%). Deepseek-R1 provided a total of 6240 correct responses, while the oncologists provided 5489. This difference was statistically significant (P = 0.004) (see **Figure 2A** and **Figure 3A**).

**Table 2:** Inter-rater reliability analysis of the 64 calibration cases.

Variable	Kappa value	Asymptotic std. error	Approx. T	P-value
Doctors Group	0.613	0.088	6.530	<0.001
Ethical Review Group	0.669	0.087	7.175	<0.001

**Table 3:** Comparison of Overall Performance Between Deepseek-R1 and Junior Oncologists in 256 Diagnostic Cases.

Variable	Deepseek-R1	junior doctors	P-value
Total number of questions	6656	6656	
Basic Group accuracy	1510 (98.3)	1435 (93.4)	
Advanced Group accuracy	3633 (94.6)	3030 (78.9)	
Ethical Group accuracy	1097 (85.7)	1024 (80)	
Total	6240	5489	0.004
Knowledge errors	143 (34.4)	412 (35.3)	
Logical bias	90 (21.6)	499 (42.8)	
Ethical risks	183 (44)	256 (21.9)	
Total	416	1167	<0.001

Basic Group accuracy = (Total number of correct answers in the Basic Group/Total number of questions in the Basic Group (256×6)) × 100%.

Advanced Group accuracy = (Total number of correct answers in the Advanced Group/Total number of questions in the Advanced Group (256×15)) × 100%.

Ethical Group patterns = (Total number of correct answers in the Ethical Group/Total number of questions in the Ethical Group (256×5)) × 100%.

Knowledge errors proportion = (Knowledge errors in Basic Group + Advanced Group)/Total number of errors (Total questions - Total correct answers) × 100%.

Logical bias proportion = (Logical bias in Advanced Group)/Total number of errors (Total questions - Total correct answers) × 100%.

Ethical risks = (Ethical risks in Ethical Group)/Total number of errors (Total questions - Total correct answers) × 100%.

The results of error type analysis are shown in **Table 4**. Deepseek-R1 made 416 errors, resulting in an overall error rate of 6.2% (95% CI: 5.7% - 6.9%). In contrast, the less experienced doctors made 1167 errors, corresponding to an overall error rate of 17.5% (95% CI: 16.6% - 18.5%). The model exhibited significantly fewer errors in both total number and error rate. The incidence rates of specific error types, expressed as a percentage of the total number of questions, are as follows: For knowledge-based errors, Deepseek-R1 had an incidence of 2.1% (95% CI: 1.8% - 2.5%), while the doctors had a rate of 6.2% (95% CI: 5.6% - 6.8%). For logical errors, Deepseek-R1 showed a rate of 1.4% (95% CI: 1.1% - 1.7%), whereas the doctors exhibited a rate of 7.5% (95% CI: 6.9% - 8.2%). For ethical errors, Deepseek-R1 had a rate of 2.7% (183/6656, 95% CI: 2.4% - 3.2%), while the doctors had a rate of 3.8% (95% CI: 3.4% - 4.3%).Although the proportion of ethical errors in total errors was higher in the model (44.0%) than in the doctors (21.9%), the absolute rate of ethical errors was lower in the model (2.7%) compared to the doctors (3.8%). Overall, Deepseek-R1 demonstrated superior performance, particularly with respect to knowledge-based and logical errors, maintaining a lower overall error rate and showing stronger logical reasoning and decision-making consistency (see **Figure 2B** and **Figure 3B**).

**Table 4:** Longitudinal simulation of diagnostic performance between deepseek-r1 and oncologists in 40 progressive-phase lung cancer cases.

Variable	Deepseek-R1	junior doctors	P-value
Total number of questions	360	360	
Basic Group accuracy	116 (96.7)	113 (94.2)	
Advanced Group accuracy	114 (95)	86 (71.7)	
Ethical Group accuracy	103 (85.8)	98 (81.7)	
Total	333	297	0.667
Knowledge errors	6 (22.2)	10 (15.9)	
Logical bias	4 (14.8)	30 (47.6)	
Ethical risks	17 (63)	23 (36.5)	
Total	27	63	0.012

Basic Group accuracy = (Total number of correct answers in the Basic Group/Total number of questions in the Basic Group (120)) × 100%.

Advanced Group accuracy = (Total number of correct answers in the Advanced Group/Total number of questions in the Advanced Group (120)) × 100%.

Ethical Group patterns = (Total number of correct answers in the Ethical Group/Total number of questions in the Ethical Group (120)) × 100%.

Knowledge errors proportion = (Knowledge errors in Basic Group + Advanced Group)/Total number of errors (Total questions - Total correct answers) × 100%.

Logical bias proportion = (Logical bias in Advanced Group)/Total number of errors (Total questions - Total correct answers) × 100%.

Ethical risks = (Ethical risks in Ethical Group)/Total number of errors (Total questions - Total correct answers) × 100%.

**Table 5** shows the longitudinal simulation results for 40 patients in the advanced stage. After dynamically inputting the three-phase information (baseline → treatment → progression), Deepseek-R1 achieved an overall accuracy of 92.5% (95% CI: 89.3% - 94.8%), while the doctors achieved an accuracy of 82.5% (95% CI: 78.2% - 86.1%). In the advanced group, the model’s accuracy was 95.0% (95% CI: 89.5% - 97.7%), significantly higher than the doctors’ 71.7% (95% CI: 63.0% - 79.0%). Error rate analysis of 360 questions showed that Deepseek-R1 had an overall error rate of 7.5% (95% CI: 5.2% - 10.7%), whereas the doctors had a rate of 17.5% (95% CI: 13.9% - 21.8%). For logical errors, Deepseek-R1 had a rate of 1.1% (95% CI: 0.4% - 2.8%), compared to 8.3% (95% CI: 5.9% - 11.6%) for the doctors. Although the proportion of logical errors in total errors was higher for the doctors (47.6%) than for the model (14.8%), this difference was statistically significant (P = 0.012). However, the error rate for logical errors was significantly lower in the model. These findings further validate the stability of the model’s reasoning in dynamic tasks (see **Figures 3C, D**).

**Table 5:** 26 questions concerning the Fundamental Group, Advanced Group, and Ethical Group.

Fundamental group	
Question 1	What is the definition of lung cancer?
Question 2	What are the common etiological factors of lung cancer?
Question 3	Based on the CT imaging data of this patient, is a diagnosis possible?
Question 4	Where is the lesion located according to the CT imaging findings?
Question 5	What further examinations should be conducted after a suspected diagnosis of lung cancer?
Question 6	How should the treatment plan be developed based on the patient’s tumor classification and staging?

Advanced group	
Question 1	How should the subsequent treatment plan be adjusted based on the patient’s pathology report (e.g., adenocarcinoma, squamous carcinoma, or small cell carcinoma)?
Question 2	Based on the TNM staging system, how should the lung cancer stage of this patient be accurately classified? What imaging or pathological features should be considered?
Question 3	For a lung mass with ill-defined borders and no typical spiculated signs on CT imaging, what differential diagnoses should be prioritized?
Question 4	How can the likelihood of mediastinal lymph node metastasis be assessed? What additional imaging or laboratory tests should be performed?
Question 5	If imaging suggests peripheral lung cancer but the pathology results show a benign lesion, how should the diagnostic process be reassessed?
Question 6	How can surgical or chemotherapy plans be optimized for lung cancer patients with concurrent COPD or cardiovascular disease?
Question 7	How should targeted therapy be selected based on the patient’s genetic testing results (e.g., EGFR mutations)?
Question 8	For a patient with advanced non-small cell lung cancer (NSCLC) and a PD-L1 expression level of 30%, is immune checkpoint inhibitor therapy recommended?
Question 9	For post-operative stage IIIA lung cancer patients, what is the optimal timing and dosage for combined radiotherapy and chemotherapy?
Question 10	For pain management in terminal-stage lung cancer patients, which drug combinations should be prioritized?
Question 11	If acquired resistance to EGFR-TKI occurs during treatment, what are the subsequent treatment options?
Question 12	How should severe bone marrow suppression after chemotherapy be managed, and how should the subsequent treatment plan be adjusted?
Question 13	For lung cancer patients with brain metastases, is a consultation with neurosurgery or radiation oncology necessary?
Question 14	If new liver metastases appear after chemotherapy, how should the original treatment plan be adjusted?
Question 15	How should rare pathological types, such as pulmonary sarcomatoid carcinoma, be diagnosed? What specific tests are needed?

Ethical group	
Question 1	How should the 5-year survival rate for stage III B lung cancer be explained to the patient and their family? What communication skills should be prioritized?
Question 2	For patients with advanced PD-L1-negative tumors, are there any eligible clinical trials that can be recommended?
Question 3	In cases with a limited budget, how can cost-effective diagnostic and therapeutic options be prioritized?
Question 4	If the model initially diagnoses “inflammation” but the pathology results show “malignancy,” can the diagnosis be autonomously corrected, and how should the reasoning be explained?
Question 5	How can the privacy and security of imaging and genetic testing data provided by patients be ensured during model interactions?

**Table 5**: Regarding the 26 questions raised.

## Discussion

In recent years, the application of artificial intelligence (AI) in the medical field has expanded (17), especially in the diagnosis and treatment of high-incidence diseases like lung cancer, where it has shown significant potential. AI leverages core technologies, including big data analysis, deep learning, and natural language processing, to assist physicians in making accurate diagnoses and to provide essential support in treatment decisions, personalized plan development, and prognosis evaluation. Large language models (LLMs), capable of understanding and integrating vast amounts of medical literature, clinical guidelines, and real-world case data, are increasingly becoming crucial tools for medical knowledge retrieval and clinical reasoning. Compared to traditional AI systems, LLMs offer superior cross-task transfer capabilities and enhanced natural language interaction, allowing them to provide real-time knowledge support and decision-making assistance to clinicians with minimal complexity. Existing studies have demonstrated that AI technology has been successfully applied to various medical tasks, including measuring pancreatic cystic lesions, identifying metastatic disease locations, and extracting oncology-related outcomes from free-text or semi-structured reports (8, 18–20). These achievements further validate the vast potential of AI in improving the efficiency and accuracy of medical diagnostics.

This study evaluates the accuracy of the Deepseek-R1 model in processing information for lung cancer patients. In clinical practice, the model’s responses to 26 questions were generally satisfactory, particularly when addressing conceptual queries, where its answers were both accurate and comprehensive. This finding is consistent with that of Butler et al (21), who assessed the performance of AI large language models (AI-LLMs) in enhancing the readability of ankle radiology reports. Their study graded the generated reports based on readability and accuracy, utilizing the Flesch Reading Ease Score (FRES) and Flesch-Kincaid Reading Level (FKRL) as evaluation metrics. The results indicated that AI-LLMs significantly improved report readability while maintaining high accuracy. Additionally, in clinical diagnostics, the Deepseek-R1 model achieved an accuracy rate of 94.6%, substantially outperforming less experienced physicians, who achieved an accuracy of 78.9%. This outcome is consistent with previous studies. For instance, Do et al (20) evaluated the potential of natural language processing (NLP) in extracting metastatic disease information from CT imaging reports of cancer patients. Their comprehensive assessment of the NLP model’s performance, using metrics such as accuracy, precision, and recall, was based on 387,000 reports from over 90,000 patients. The study developed three NLP models to predict the presence of metastatic disease in 13 organs, with the best-performing model achieving an accuracy of 90%-99% across all organs. Furthermore, the study identified specific metastatic patterns for different cancer types, such as breast and prostate cancers, which tend to metastasize to bones, while colorectal and pancreatic cancers preferentially metastasize to the liver. This research demonstrated the significant potential of NLP in building large-scale metastatic disease databases and highlighted its importance in personalized treatment planning. This finding parallels our study, in which we observed that more complex disease conditions in 40 patients with advanced lung cancer led to a sharp decline in diagnostic accuracy among less experienced physicians, particularly when clinical logic deviations reached 47.6%. Despite this, the Deepseek-R1 model maintained a stable accuracy rate in clinical diagnostic tasks. Although our study did not track or quantify how Deepseek-R1 dynamically adjusts recommendations based on longitudinal case updates, its high accuracy in handling complex cases with disease progression information suggests its potential adaptability. For instance, when faced with a patient previously diagnosed with an EGFR-sensitive mutation in non-small cell lung cancer, following radiological progression after first-generation TKI treatment and the detection of the T790M resistance mutation, Deepseek-R1 could potentially recognize the resistance mechanism based on updated clinical information and recommend a treatment switch to osimertinib. This ability to adjust treatment strategies based on key clinical milestones, such as newly detected resistance mutations or significant changes in comorbidities, is a crucial aspect of assessing AI’s clinical value. Future research should focus on developing more refined evaluation methods to capture and validate the dynamic adjustment process of AI recommendations as patient conditions evolve, particularly in real or highly simulated longitudinal case management, and assess the clinical rationale behind these adjustments. This could involve documenting the model’s specific responses after receiving updated information at various time points and evaluating their consistency with clinical guidelines and expert consensus. In clinical diagnostics, Deepseek-R1 occasionally exhibits logical deviations and generates “hallucinations,” or fabricated neutral facts (22). Karan et al. (23) noted that approximately 30% of LLM outputs were inconsistent with medical facts or consensus. Given the limited experience of junior physicians and the long-term expertise required for lung cancer diagnosis and treatment decision-making, Deepseek-R1’s suggestions still provide valuable support to junior doctors. However, final diagnostic decisions should be compared and confirmed with the conclusions of senior oncologists. Our study comprehensively evaluated the Deepseek-R1 model in the context of lung cancer, addressing basic concepts, clinical diagnostics, and ethical considerations. The results indicated that the model demonstrated high accuracy across various aspects. However, our findings diverged from those of other studies, which showed shortcomings in certain characteristics and question categories, particularly in terms of consistency and evidence-based performance (24). We believe that these discrepancies may be attributed to the detailed patient diagnostic information provided in our study. While AI shows promise in disease diagnosis, it still has significant limitations, particularly in the following areas: First, disease diagnosis requires multiple types of information, including detailed medical history, physical examination, imaging tests (such as CT and MRI), and laboratory tests. However, AI cannot access real-time dynamic patient information, such as the specific location and severity of symptoms, associated symptoms, and their progression. Additionally, AI cannot perform physical exams, such as palpation, percussion, or reflex testing, which are critical for clinicians to make intuitive judgments about a patient’s pathological state. Finally, imaging and laboratory test results are often essential for diagnosing diseases, assessing their severity, and formulating treatment plans. Since AI relies solely on limited descriptive information and imaging data, the singularity and incompleteness of these inputs directly limit the accuracy and comprehensiveness of the diagnosis. Second, medical diagnosis requires highly individualized analysis, especially when significant differences exist among patients. Current AI models often lack a deep understanding of a patient’s overall health, potential comorbidities, and individual differences, making it challenging to provide precise treatment recommendations. In conclusion, the limitations of the Deepseek-R1 model primarily lie in its lack of comprehensive patient data, its inability to perform clinical exams, and its absence of individualized analysis capabilities.

With the gradual integration of artificial intelligence (AI) into clinical practice, several concerns have emerged. First, particular attention must be paid to the accuracy and clinical validity of the generated content, as incorrect information could mislead patients and even pose risks. While the Deepseek-R1 model is capable of providing extensive medical knowledge and supporting clinical decision-making to some degree, it cannot yet fully replace the judgment of professional healthcare providers in all contexts. Unlike traditional search engines, Deepseek-R1 does not provide explicit sources for the information it generates; instead, it produces responses based on a vast pool of data. Although AI has made significant strides in the recognition and classification of digital pathology images, and certain visual models have demonstrated high accuracy in clinical diagnostic assistance, the Deepseek-R1 model used in this study is a purely text-based model that lacks native image understanding capabilities. In this study, all imaging and pathology results were converted into standardized textual inputs by clinical doctors or optical character recognition (OCR) tools, which restricted the model’s performance to text-based data. Therefore, to achieve a higher level of “visual-language” collaborative reasoning, the development of multimodal model architectures and integration strategies with native capabilities is required. Although Deepseek-R1 can generate reasonable inferences based on existing medical knowledge, it remains limited in its ability to process complex visual cues and contextual understanding necessary for clinical diagnostics. For instance, while Deepseek-R1 can recommend antipyretic drugs for patients with common symptoms, such as fever, it cannot accurately identify the presence of infection, rashes, or other potential causes, which may result in incorrect diagnoses or delayed treatments (25). Second, the widespread application of AI in medical diagnosis has raised significant ethical and privacy concerns. During interactions with the Deepseek-R1 model, patients may provide sensitive health data, including personal information, medical conditions, and even images related to sensitive areas. A report by Lu et al (26) specifically highlighted that adolescents and young adults diagnosed with cancer face an increased suicide risk during the first year post-diagnosis. Although the Deepseek-R1 model demonstrates strong overall diagnostic and treatment recommendations for lung cancer, it exhibits a high proportion of ethical-related errors (44%), notably higher than the 21.9% observed in the physician group. This discrepancy reflects its clear limitations in non-structured tasks, such as emotional communication and value judgment. Furthermore, the model is unable to provide the emotional support or humanistic comfort required by patients. Amir et al. (27) reported instances in which the model incorrectly stated survival data for Lung-RADS, claiming that the survival time for Lung-RADS 4A was 12–18 months, and for Lung-RADS 4X, it was 3–6 months, which contradicted actual guidelines. The dissemination of such erroneous information could not only mislead patients but also result in significant emotional distress, with potentially catastrophic consequences. Therefore, it is advisable to avoid having the model directly provide patients with highly sensitive information, such as survival estimates. Further analysis revealed that when interacting with cancer patients, Deepseek-R1 often describes disease progression and survival timelines in a blunt manner, without contextual adjustment, thereby adding psychological and emotional burdens on the patients. In contrast, although junior doctors made more ethical-related errors than Deepseek-R1 in absolute terms, these errors were primarily due to inadequate communication skills, which are more related to limited clinical experience. This suggests that, while AI models may outperform human doctors in certain judgment tasks, they still have significant limitations in humanistic care, ethical judgment, and communication. These ethical-related errors manifest in multiple forms. For example, in simulated clinical dialogues, Deepseek-R1 sometimes recommends invasive procedures or high-risk treatments without adequately explaining or warning about potential risks, possibly overlooking the patient’s right to informed consent. Additionally, when handling cases involving sensitive personal information, even though the data is initially anonymized, the model occasionally infers or suggests details that could inadvertently reveal the patient’s identity when generating summary reports or responding to follow-up inquiries, raising concerns about patient privacy protection. Moreover, the model has been observed to exhibit potential biases when confronted with ethical dilemmas related to resource allocation or clinical trial recommendations. For example, when recommending clinical trials, it may fail to consider the accessibility differences among patients from varying socioeconomic backgrounds, or in some instances, its suggestions may unintentionally favor certain populations. This suggests the presence of bias in the model’s decision-making logic. These ethical challenges underscore the need for more thorough ethical review and algorithmic optimization before applying such large language models in real-world clinical decision support.

In this study, we conducted a preliminary analysis of the “hallucination phenomenon” in the Deepseek-R1 model for lung cancer tasks. Hallucination is defined as the generation of information that appears plausible but is inconsistent with facts or medical consensus. We found that Deepseek-R1 occasionally generated fictitious values, guideline entries, or recommendation pathways in the absence of clear input support. For example, the model erroneously cited non-existent drug usage suggestions or fabricated causal relationships between certain pathological subtypes and specific mutations. These errors were particularly common in cases involving ethical issues, manifesting as unverified treatment pathways or unrealistic survival expectations. Although we have not yet systematically quantified the frequency of these errors, our experiments suggest that this hallucination phenomenon is highly concerning. In structured tasks, the model achieved an overall accuracy of 96.2% in the basic and advanced groups, significantly higher than the physician group’s 86.5%. This highlights the model’s strong transferability and consistency. However, while the model can generate diagnostic results in complex scenarios, it still falls short of experienced clinicians in identifying misdiagnosis risks, integrating clinical context, and providing individualized explanations. As Berry et al. (28) noted in their study on lung ultrasound, the acquisition and interpretation of LUS images are highly dependent on the operator’s experience and professional judgment. Even after short-term training, operators may misjudge complex lesions or artifacts. Similarly, current AI diagnostic systems face limitations in complex situations requiring the integration of multi-source clinical information and differential diagnosis. In such cases, AI cannot achieve the flexible adjustment and individualized decision-making capabilities of physicians. We further analyzed the error causes in the basic and advanced groups of Deepseek-R1. In the basic group, the model primarily made knowledge-based errors in identifying common risk factors for lung cancer and in initial judgments of CT imaging localization. These errors may stem from insufficient medical specificity and clinical diversity in the pre-training data. In the advanced group, logical bias was the main type of error, with typical mistakes occurring in complex TNM staging judgments and treatment pathway adjustments following resistance mutations (e.g., after EGFR-TKI treatment). This highlights the model’s limitations in handling multi-parameter comprehensive reasoning tasks, particularly when dealing with rare pathological subtypes, such as pulmonary sarcomatoid carcinoma. Compared to junior doctors, Deepseek-R1 exhibited lower rates of knowledge-based errors (2.1%) and logical errors (1.4%), while junior doctors had error rates of 6.2% and 7.5%, respectively. This suggests that in tasks requiring up-to-date and detailed knowledge, such as accurately classifying uncommon lung cancer subtypes (e.g., sarcomatoid carcinoma) or identifying rare driver genes (e.g., precise identification of ROS1 fusion and its association with ALK-negative status), junior doctors may be more prone to knowledge-based biases due to insufficient clinical experience or incomplete knowledge updates. Similarly, junior doctors have a higher rate of logical errors in cases involving complex comorbidities, which may reflect an imperfect logical chain in information integration, risk assessment, and individualized decision-making. For example, formulating a treatment plan for a patient with advanced lung cancer and severe cardiopulmonary insufficiency poses significant challenges to logical reasoning, as it requires balancing the intensity of anti-tumor treatment with patient tolerance and estimating the potential risks of treatment-related complications. While the proportion of ethical errors in Deepseek-R1’s error composition is relatively high (44.0% of errors), its absolute rate of ethical errors (2.7%) remains lower than that of physicians (3.8%). The model also demonstrates advantages in terms of broad knowledge coverage, rapid information updates, and logical consistency under rule-based scenarios. However, AI models still face inherent limitations. For example, the model may encounter knowledge gaps or rigid logic when dealing with the latest research details of “rare mutations” not fully covered in the training data or complex comorbidity management strategies requiring high individualization beyond conventional guidelines. In such cases, the model may fail to provide optimal suggestions. Future research should focus on in-depth analysis of AI error patterns in specific clinical challenges and explore improvement strategies. These strategies could include fine-tuning with more rare case data or enhancing the model’s reasoning capabilities for handling multi-source uncertain information, in order to better support clinical decision-making.

Artificial intelligence (AI) has the potential to provide accurate and effective clinical support, but it may also lead to over-reliance in the medical field. This concern is well-founded, as multiple studies and commentaries have highlighted the potential negative impacts of AI. For example, the US National Institute of Standards and Technology (NIST) reported that human cognitive biases, such as automation bias or over-reliance on automated systems, can diminish critical thinking in AI-assisted decision-making. Human biases often reflect systematic errors in thinking associated with heuristic principles and predictive values linked to simple judgment operations. These biases are typically implicit and influence how individuals or groups perceive information, such as automated AI output, to make decisions or fill in missing information. As a result, healthcare professionals who overly trust AI outputs may unconsciously reduce their independent critical assessment, particularly in complex or ambiguous situations, potentially compromising decision quality (29). Similarly, Siala et al. (30)emphasized the importance of responsible AI use in healthcare and highlighted the risks associated with over-reliance on AI. Over-reliance on AI systems for diagnosis and decision-making can erode healthcare professionals’ clinical skills, critical thinking, and local practice capabilities. Specifically, physicians may gradually lose their ability to independently assess complex cases, particularly when conducting detailed differential diagnoses or making flexible judgments based on individual factors. This directly impacts the quality and safety of medical services. While language models have advantages in processing and analyzing large-scale medical data and can support clinical decision-making, their functions are limited to data-driven pattern recognition and predictive analysis. They lack a deep understanding of potential variables in complex medical contexts and the integration of clinical experience. Therefore, AI should be viewed as a complementary tool to medical expertise, rather than a substitute for the core judgment and decision-making roles of physicians. Physicians’ experience, clinical knowledge, ethical considerations, and patient individual differences remain essential factors in the decision-making process. Moreover, AI language models are trained on large datasets, which may introduce biases inherent in the data. If the training data are biased or erroneous, the model may inadvertently propagate these biases or errors in its outputs. Therefore, careful selection of training data and ongoing bias detection and correction efforts are essential to ensure the reliability and accuracy of AI models.

Although the Deepseek-R1 model showed high accuracy and clinical utility in generating diagnostic and therapeutic recommendations for lung cancer, several limitations hinder its broader implementation. First, the model’s performance depends heavily on the quality and completeness of input data. In this study, experienced oncologists verified all patient records to ensure accuracy and consistency. However, consistent data quality cannot be guaranteed in real-world clinical environments. The model’s robustness in scenarios involving incomplete information or complex comorbidities remains insufficiently validated. Although expert verification enhances data reliability, the structured inputs used in this study fail to capture the uncertainty and variability typically present in routine clinical documentation. Moreover, converting multimodal clinical data into plain text inevitably leads to information loss. For example, converting radiological data such as CT scans into standardized text often omits critical features—such as lesion texture, margins, vascular involvement, and spatial anatomy. These features serve as key diagnostic cues for experienced clinicians. This abstraction may limit the model’s capacity to capture the full clinical context required for managing complex cases. To address these limitations, future work should prioritize developing multimodal large language models (LLMs) that integrate both visual and textual clinical information. These models could improve AI interpretability and robustness by enabling cross-modal reasoning in clinical applications. Additionally, to assess the model’s tolerance for flawed inputs and its error-correction capability, future studies should include test sets containing misleading or contradictory clinical data. Such “adversarial” or “conflict simulation” sets could be used to evaluate the model’s capacity for discrimination and self-correction under diagnostic ambiguity. Prompt engineering strategies should also be explored. For example, prompts including verification cues—such as “Please confirm whether this recommendation aligns with guidelines”—may enhance output reliability and robustness in response to atypical or noisy inputs. This study did not perform subgroup sensitivity analyses, introducing a potential bias due to imbalanced patient subgroup distribution. The single-center retrospective design and limited sample size may have introduced selection bias. Future validation should use multicenter, large-scale datasets and include stratified subgroup analyses to evaluate the model’s generalizability and stability across clinical contexts. Finally, the current model cannot accommodate real-time clinical changes or patient-specific disease trajectories. To overcome this, future models should incorporate time-series frameworks and integrate heterogeneous data sources—including genomics, transcriptomics, and electronic medical records. This approach would enable dynamic modeling of disease progression and enhance personalized medicine. Technically, feedback mechanisms linked to disease progression could enable adaptive output updates and help overcome the limitations of rule-based systems in managing non-linear and stage-dependent disease trajectories.

## Conclusion

Deepseek-R1 significantly outperformed junior oncologists in terms of diagnostic accuracy and treatment decision-making, particularly in complex and dynamic clinical situations. While limitations remain in its ethical reasoning, the model holds substantial potential for supporting junior physicians, contributing to multidisciplinary discussions, and optimizing treatment pathways.

## Data Availability

The raw data supporting the conclusions of this article will be made available by the authors, without undue reservation.

## References

[1] Klotz R Pausch TM Kaiser J Joos MC Hecktor R Ahmed A. ChatGPT vs. surgeons on pancreatic cancer queries: accuracy & empathy evaluated by patients and experts. HPB (Oxford). (2025) 27:311–7. doi: 10.1016/j.hpb.2024.11.012, PMID: 39672696

[2] McKinney SM Sieniek M Godbole V Godwin J Antropova N Ashrafian H. International evaluation of an AI system for breast cancer screening. Nature. (2020) 577:89–94. doi: 10.1038/s41586-019-1799-6, PMID: 31894144

[3] Kuckelman IJ Wetley K Yi PH Ross AB. Translating musculoskeletal radiology reports into patient-friendly summaries using ChatGPT-4. Skeletal Radiol. (2024) 53:1621–4. doi: 10.1007/s00256-024-04599-2, PMID: 38270616

[4] Tu Z Talebi H Zhang H Yang F Milanfar P Bovik A. Maxvit: Multi-axis vision transformer. In: Paper presented at: European conference on computer vision (2022).

[5] Chen T Saxena S Li L Fleet DJ Hinton G. Pix2seq: A language modeling framework for object detection. arXiv preprint arXiv. (2021), 210910852. doi: 10.48550/arXiv.2109.10852

[6] Chowdhery A Narang S Devlin J Bosma M Mishra G Roberts A. Palm: Scaling language modeling with pathways. J Mach Learn Res. (2023) 24:1–113. doi: 10.48550/arXiv.2204.02311

[7] Zhao WX Zhou K Li J Tang T Wang X Hou Y. A survey of large. Lang models. arXiv preprint arXiv. (2023), 230318223. doi: 10.48550/arXiv.2303.18223

[8] Fink MA Bischoff A Fink CA Moll M Kroschke J Dulz L. Potential of chatGPT and GPT-4 for data mining of free-text CT reports on lung cancer. Radiology. (2023) 308:e:231362. doi: 10.1148/radiol.231362, PMID: 37724963

[9] Mesko B. The chatGPT (Generative artificial intelligence) revolution has made artificial intelligence approachable for medical professionals. J Med Internet Res. (2023) 25:e48392. doi: 10.2196/48392, PMID: 37347508 PMC10337400

[10] Shea YF Lee CMY Ip WCT Luk DWA Wong SSW. Use of GPT-4 to analyze medical records of patients with extensive investigations and delayed diagnosis. JAMA Netw Open. (2023) 6:e2325000. doi: 10.1001/jamanetworkopen.2023.25000, PMID: 37578798 PMC10425828

[11] Sarraju A Bruemmer D Van Iterson E Cho L Rodriguez F Laffin L. Appropriateness of cardiovascular disease prevention recommendations obtained from a popular online chat-based artificial intelligence model. JAMA. (2023) 329:842–4. doi: 10.1001/jama.2023.1044, PMID: 36735264 PMC10015303

[12] Haver HL Ambinder EB Bahl M Oluyemi ET Jeudy J Yi PH. Appropriateness of breast cancer prevention and screening recommendations provided by chatGPT. Radiology. (2023) 307:e230424. doi: 10.1148/radiol.230424, PMID: 37014239

[13] Henson JB Glissen Brown JR Lee JP Patel A Leiman DA. Evaluation of the potential utility of an artificial intelligence chatbot in gastroesophageal reflux disease management. Am J Gastroenterol. (2023) 118:2276–9. doi: 10.14309/ajg.0000000000002397, PMID: 37410934 PMC10834834

[14] Peng W Feng Y Yao C Zhang S Zhuo H Qiu T. Evaluating AI in medicine: a comparative analysis of expert and ChatGPT responses to colorectal cancer questions. Sci Rep. (2024) 14:2840. doi: 10.1038/s41598-024-52853-3, PMID: 38310152 PMC10838275

[15] Pandya S Bresler TE Wilson T Htway Z Fujita M. Decoding the NCCN guidelines with AI: A comparative evaluation of chatGPT-4. 0 Llama 2 Manage Thyroid Carcinoma. Am Surg. (2025) 91:94–8. doi: 10.1177/00031348241269430, PMID: 39136578

[16] Gibney E. China’s cheap, open AI model DeepSeek thrills scientists. Nature. (2025) 638:13–4. doi: 10.1038/d41586-025-00229-6, PMID: 39849139

[17] Mastrokostas PG Mastrokostas LE Emara AK Wellington IJ Ginalis E Houten JK. GPT-4 as a source of patient information for anterior cervical discectomy and fusion: A comparative analysis against google web search. Global Spine J. (2024) 14:2389–98. doi: 10.1177/21925682241241241, PMID: 38513636 PMC11529100

[18] Yamashita R Bird K Cheung PY Decker JH Flory MN Goff D. Automated identification and measurement extraction of pancreatic cystic lesions from free-text radiology reports using natural language processing. Radiol Artif Intell. (2022) 4:e210092. doi: 10.1148/ryai.210092, PMID: 35391762 PMC8980879

[19] Bozkurt S Alkim E Banerjee I Rubin DL. Automated detection of measurements and their descriptors in radiology reports using a hybrid natural language processing algorithm. J Digit Imaging. (2019) 32:544–53. doi: 10.1007/s10278-019-00237-9, PMID: 31222557 PMC6646482

[20] Do RKG Lupton K Causa Andrieu HPI Luthra A Taya M Batch K. Patterns of metastatic disease in patients with cancer derived from natural language processing of structured CT radiology reports over a 10-year period. Radiology. (2021) 301:115–22. doi: 10.1148/radiol.2021210043, PMID: 34342503 PMC8474969

[21] Butler JJ Harrington MC Tong Y Rosenbaum AJ Samsonov AP Walls RJ. From jargon to clarity: Improving the readability of foot and ankle radiology reports with an artificial intelligence large language model. Foot Ankle Surg. (2024) 30:331–7. doi: 10.1016/j.fas.2024.01.008, PMID: 38336501

[22] Shen Y Heacock L Elias J Hentel KD Reig B Shih G. ChatGPT and other large language models are double-edged swords. Radiology. (2023) 307:e230163. doi: 10.1148/radiol.230163, PMID: 36700838

[23] Singhal K Azizi S Tu T Mahdavi SS Wei J Chung HW. Large language models encode clinical knowledge. arXiv preprint arXiv. (2022), 221213138. doi: 10.48550/arXiv.2212.13138 PMC10396962 37438534

[24] Magruder ML Rodriguez AN Wong JCJ Erez O Piuzzi NS Scuderi GR. Assessing ability for chatGPT to answer total knee arthroplasty-related questions. J Arthroplasty. (2024) 39:2022–7. doi: 10.1016/j.arth.2024.02.023, PMID: 38364879

[25] Xue VW Lei P Cho WC. The potential impact of ChatGPT in clinical and translational medicine. Clin Transl Med. (2023) 13:e1216. doi: 10.1002/ctm2.1216, PMID: 36856370 PMC9976604

[26] Lu D Fall K Sparen P Ye W Adami H-O Valdimarsdóttir U. Suicide and suicide attempt after a cancer diagnosis among young individuals. Ann Oncol. (2013) 24:3112–7. doi: 10.1093/annonc/mdt415, PMID: 24169626

[27] Rahsepar AA Tavakoli N Kim GHJ Hassani C Abtin F Bedayat A. How AI responds to common lung cancer questions: chatGPT vs google bard. Radiology. (2023) 307:e230922. doi: 10.1148/radiol.230922, PMID: 37310252

[28] Berry L Rehnberg L Groves P Knight M Stewart M Dushianthan A. Lung ultrasound in critical care: A narrative review. Diagnostics (Basel). (2025) 15. doi: 10.3390/diagnostics15060755, PMID: 40150097 PMC11941729

[29] Schwartz R Vassilev A Greene K Perine L Burt A Hall P. (2022).

[30] Siala H Wang Y. SHIFTing artificial intelligence to be responsible in healthcare: A systematic review. Soc Sci Med. (2022) 296:114782. doi: 10.1016/j.socscimed.2022.114782, PMID: 35152047

