# Supplementary material for: The clinical application potential assessment of the Deepseek-R1 large language model in lung cancer
Source: Front Oncol. 2025 Sep 2;15:1601529. doi: 10.3389/fonc.2025.1601529 (PMC12436400; doi:10.3389/fonc.2025.1601529)
Supplement: Supplementary file 1 [file Table1.docx]

# Appendix A. JSON Input and Text Standardization Examples

## Appendix A. De-identified JSON Input Example

| Field Name | Example Value |
| --- | --- |
| patient_id | P013 |
| age | 64 |
| gender | male |
| smoking_history | current smoker, 40 pack-years |
| symptoms | persistent cough, mild hemoptysis |
| ct_report | A 2.5 cm spiculated nodule in the right upper lobe, no pleural effusion. |
| pathology | moderately differentiated adenocarcinoma |
| genetics.EGFR | positive |
| genetics.ALK | negative |

## Appendix B. Original Medical Description vs. Standardized Input

| Original Description | Standardized Input |
| --- | --- |
| A 2.5 cm spiculated nodule in the right upper lobe, with unclear borders and no pleural effusion. | CT scan shows a 2.5 cm spiculated nodule in the right upper lobe. No pleural effusion. |
